# The nucleosome regulates the usage of polyadenylation sites in the human genome

**DOI:** 10.1186/1471-2164-14-912

**Published:** 2013-12-23

**Authors:** Huan Huang, Jiao Chen, Hongde Liu, Xiao Sun

**Affiliations:** 1 State Key Laboratory of Bioelectronics, School of Biological Science and Medical Engineering, Southeast University, Nanjing 210096, China

## Abstract

**Background:**

It has been reported that 3*'* end processing is coupled to transcription and nucleosome depletion near the polyadenylation sites in many species. However, the association between nucleosome occupancy and polyadenylation site usage is still unclear.

**Results:**

By systematic analysis of high-throughput sequencing datasets from the human genome, we found that nucleosome occupancy patterns are different around the polyadenylation sites, and that the patterns associate with both transcription termination and recognition of polyadenylation sites. Upstream of proximal polyadenylation sites, RNA polymerase II accumulated and nucleosomes were better positioned compared with downstream of the sites. Highly used proximal polyadenylation sites had higher upstream nucleosome levels and RNA polymerase II accumulation than lowly used sites. This suggests that nucleosomes positioned upstream of proximal sites function in the recognition of proximal polyadenylation sites and in the preparation for 3*'* end processing by slowing down transcription speed. Both conserved distal polyadenylation sites and constitutive sites showed stronger nucleosome depletion near polyadenylation sites and had intrinsically better positioned downstream nucleosomes. Finally, there was a higher accumulation of RNA polymerase II downstream of the polyadenylation sites, to guarantee gene transcription termination and recognition of the last polyadenylation sites, if previous sites were missed.

**Conclusions:**

Our study indicates that nucleosome arrays play different roles in the regulation of the usage of polyadenylation sites and transcription termination of protein-coding genes, and form a dual pausing model of RNA polymerase II in the alternative polyadenylation sites’ region, to ensure effective 3*'* end processing.

## Background

Formation of the 3′ end of precursor messenger RNA (pre-mRNA) is an essential step in the procedure of eukaryotic gene expression. Inappropriate 3′ end formation of human mRNAs can have a tremendous impact on health and disease [1,2]; however the molecular mode of action is still unknown. 3′ End processing involves two tightly coupled steps, cleavage and polyadenylation, and requires a polyadenylation signal (PAS) and a downstream sequence element (DSE) [3]. Transcription termination is triggered following recognition of the polyadenylation signal by RNA polymerase II (RNAP II) and subsequent pre-mRNA cleavage, which occurs at the polyadenylation site (polyA site). Interestingly, it has been shown that the strength of the polyA site correlates with efficient pausing-dependent termination [4,5].

Recent evidence has indicated that 3′ end processing is coupled to transcription and splicing, as well as termination [6-8], and that RNAP II plays a critical role in coordinating co-transcriptional pre-mRNA processing [9]. The largest RNAP II subunit contains a carboxyl-terminal domain that appears to couple transcription with histone methylation, mRNA splicing and polyadenylation by mediating interactions with processing factors [10,11]. Rigo *et al*. (2005) have found that the DNA sequence is still tethered to RNAP II when the 3′ end is cleaved, and that the 3′ end processing is fast and efficient when coupled to transcription *in vitro*[12]. Glover-Cutter *et al*. (2008) have proposed a dual pausing model, where elongation arrests near the transcription start site and in the 3′ flank to allow co-transcriptional processing by factors recruited to the RNAP II ternary complex [13].

Epigenetic modifications, including DNA methylation at CpG islands [14] and H3K36 methylation [15], can influence utilization of alternative polyA sites. In addition, it has been reported that nucleosome occupancy drops precipitously near the polyadenylation site in many species [16-19]. In human T cells, nucleosome density drops dramatically near polyA sites with a canonical PAS, and highly used polyA sites have greater nucleosome occupancy in the immediate downstream region [20]. Khaladkar *et al*. (2011) have suggested that a compacted chromatin downstream of the polyA sites can slow down the elongating transcription, thus facilitating the folding of nascent mRNA in a favorable structure at the polyA site during transcription [21]. Ji *et al*. demonstrated that compared with lowly expressed genes, highly expressed genes had a lower nucleosome level around the polyA sites, indicating that transcriptional activity has an additional impact on the nucleosome level around the polyA sites [22]. Our previous studies indicated that the nucleosome level around the polyA sites is related to conservative sequence elements (PAS and DSE) [23]. Altering nucleosome density may affect both RNAP II elongation kinetics and polyadenylation, or the recruitment of the polyadenylation machinery to the nascent transcript [24,25].

Over half of the genes in the human genome have alternative polyA sites [26]. However, nucleosome regulation during the 3′ end processing is still unclear, and a detailed exploration of associations between nucleosome occupancy and pre-mRNA 3′ end formation has not been reported. In the present study, we analyzed the nucleosome distribution near the polyA sites by processing high-throughput experimental data of the human genome. Our results suggest that nucleosomes near polyA sites may have various roles in the regulation of the usage of different types of polyA sites, e.g., proximal and distal polyA sites, constitutive and alternative polyA sites. Moreover, there is a dual pausing model of RNAP II in the alternative polyA sites’ region, to ensure effective 3′ end processing.

## Results

### Nucleosomes immediately downstream of constitutive polyA sites are intrinsically better positioned

**Figure 1:**
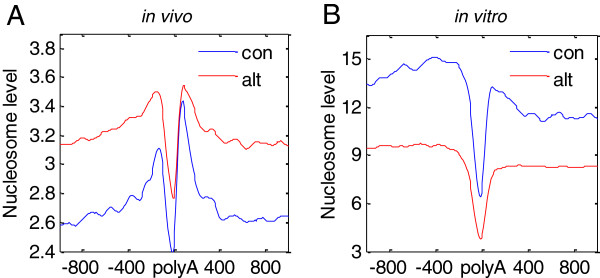
**Nucleosome distribution around constitutive and alternative polyA sites. (A)** Nucleosome occupancy surrounding constitutive (con) and alternative (alt) polyA sites across a 2000-bp window in human CD4+ T cells. **(B)** Nucleosome occupancy surrounding constitutive (con) and alternative (alt) polyA sites across a 2000-bp window *in vitro*.

Alternative cleavage and polyadenylation is a widespread phenomenon in the human genome. Chromatin structure is associated with cleavage and polyadenylation [20-23]. In the current study, to address the relationship between nucleosome occupancy and polyadenylation, the polyA sites were divided into constitutive polyA sites and alternative sites (see Methods). If a gene has only one polyA site, the site is called a constitutive site. If a gene has more than one polyA site, those sites are called alternative sites. We analyzed nucleosome distribution around constitutive and alternative polyA sites. Consistent with previous reports [18-20], nucleosome occupancy profiles showed deep troughs near polyA sites, which were observed *in vivo* and *in vitro* (Figure 1, Additional file 1A-B), indicating that this is a general phenomenon, independent of the type of cells. The fact that the *in vivo* and *in vitro* results were consistent suggests that the depleted nucleosome is partly attributed to DNA sequence. Nevertheless, the relative nucleosome level around constitutive and alternative sites was different *in vivo* and *in vitro*. Constitutive polyA sites displayed a significantly stronger nucleosome depletion near polyA sites than alternative sites did (*p* < 1 × 10^-19^, Figure 1A) *in vivo* but not *in vitro* (Figure 1B). In other types of cells, the stronger nucleosome depletion near constitutive polyA sites was also observed (Additional file 1A-B). The results suggest that nucleosome profiles around polyA sites are partly determined by the DNA sequence and cellular trans-factors, *in vivo*.

The height of the nucleosome occupancy peak downstream of constitutive polyA sites was higher than that downstream of alternative sites (Figure 1A). We quantified the significance of this difference using the F test for comparing the summit position distribution and the Student’s *t* test for comparing the fuzziness score of nucleosome peaks called by the DANPOS algorithm within 300 bp upstream and 300 bp downstream of the polyA sites for the two classes in Figure 1A (Additional file 2). The results showed that nucleosomes downstream of constitutive polyA sites were better positioned than those downstream of alternative sites. However, this difference was not significant in upstream nucleosomes (F test *p* = 0.057, *t* test *p* = 0.059).

Also, we applied the DNA sequences-base nucleosome prediction tool developed by the Segal laboratory [19], to predict nucleosome occupancy around polyA sites (Additional file 1C). The results showed that the nucleosome peak near constitutive sites is more conspicuous than that near alternative sites. These analyses confirmed our observations that nucleosomes downstream of constitutive polyA sites are intrinsically better positioned. Meanwhile, DNA sequences of alternative polyA sites are more disfavored by nucleosomes than constitutive sites. However, *in vivo*, nucleosome levels are higher, suggesting that alternative polyA sites are more affected by the *in vivo* environment and can be adjusted easily.

### The relationship between nucleosome level and the usage of constitutive and alternative polyA sites

**Figure 2:**
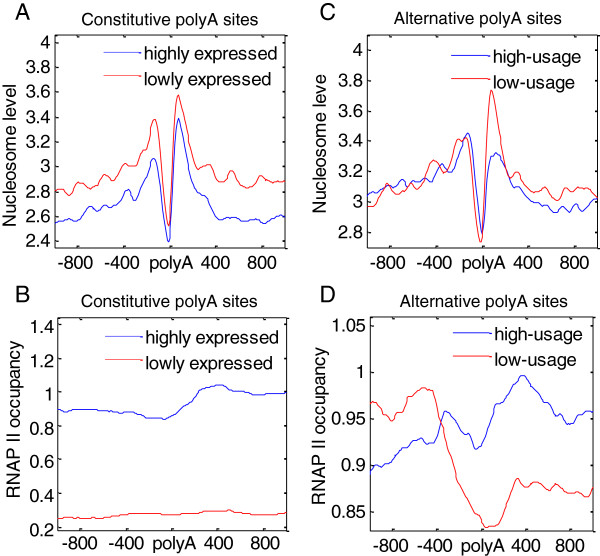
**Relationship among nucleosome, RNAP II occupancy and the usage of polyA sites. (A-B)** The nucleosome distribution and RNAP II occupancy near constitutive polyA sites of highly expressed and lowly expressed genes in CD4+ T cells (highly expressed genes: RPKM > 10, blue curve; lowly expressed genes: RPKM < 0.1, red curve). **(C-D)** The nucleosome distribution and RNAP II occupancy near the high-usage (blue curve) and low-usage (red curve) alternative polyA sites in expressed genes (RPKM > 1) in CD4+ T cells. A high-usage site has the lowest RUD in the gene, and a low-usage site has the highest RUD in the gene.

To explore the regulatory role of the nucleosome in polyA site usage, the relationship between nucleosome level and the usage of polyA site was analyzed. In the case of constitutive polyA sites, the gene expression level in the cell reflects the site usage, i.e., in highly expressed genes (RPKM > 10) the site usage is high compared with that in lowly expressed genes (RPKM < 0.1). The nucleosome level around a constitutive site negatively correlated with the gene expression, and there was a proportional relationship between RNAP II occupancy and gene expression (Figure 2A-B, Additional file 3A-B). This indicates that transcription activation can regulate nucleosome levels and that RNAP II occupancy is a determinant of nucleosome dismissing, which is consistent with a study by Ji *et al*. [22]. To directly assess whether nucleosome positioning depends on gene expression, we analyzed a set of genes differentially expressed between CD4+ T cells and granulocytes (Additional file 4). The results revealed that nucleosome level around polyA sites of the expressed genes was lower than that of the unexpressed genes *in vivo* and further confirmed that transcription activation can regulate nucleosome levels. In highly expressed genes, the reduction in downstream nucleosome levels was smaller compared with that in upstream ones, and there was a much greater accumulation of RNAP II downstream of polyA sites than upstream. Therefore, it is reasonable to postulate that a nucleosome downstream of constitutive sites is expelled by RNAP II more frequently when a gene is highly expressed, and that it is better positioned when a gene is unexpressed *in vivo*. Downstream nucleosomes may be a huge barrier for RNAP II to pass and promote transcription termination.

To evaluate alternative polyA site usage, the relative usage of downstream polyA site score (RUD) was used. Briefly, RUD is the ratio between the density of downstream reads and the density of upstream reads (see Methods). Alternative polyA sites with the lowest RUD in the gene are highly used in transcription, while those with the highest RUD in the gene are lowly used. The results showed that the changes in nucleosome levels between upstream and downstream nucleosomes were disproportional (Figure 2C, Additional file 3C-D). For high-usage alternative polyA sites, the downstream nucleosome level decreased dramatically and RNAP II occupancy increased quickly (Figure 2D), which is consistent with the constitutive sites. The change in the upstream nucleosome levels was negligible compared with that in the downstream ones. However, the RNAP II profile greatly fluctuated between high-usage alternative polyA sites and low-usage sites (Figure 2D). There was greater accumulation of RNAP II upstream of low-usage polyA sites, suggesting a different regulation of upstream nucleosomes. We conjectured that nucleosomes downstream of polyA sites are intrinsically well positioned and regulated by transcription, and upstream nucleosomes may correlate with the usage of alternative polyA sites and transcription termination. To explain this phenomenon, we performed further experiments on alternative polyA sites, which are described in the next section.

### Different nucleosome occupancy patterns around proximal and distal polyA sites

The alternative polyA sites appear in different locations, and their usage is not random. It is necessary to further explore the role of the nucleosome in the regulation of alternative polyadenylation. To this end, alternative polyA sites were divided into proximal, distal and in-between polyA sites, according to the position of the polyA site relative to the transcription start site (TSS) of the gene (see Methods). For genes with more than two polyA sites, the average distances from the three classes of polyA sites to the TSS and to the end of the gene were computed (Additional file 5). The results showed that the average distance from proximal polyA sites to the TSS of the gene is the smallest, the average distance from distal polyA sites to the end of the gene is the smallest, and the average distances from in-between sites to the TSS and to the end of the gene are in between. The differences among the average distances from the three classes to the TSS and to the end of the gene were far more than the length of nucleosome DNA (147 bp). Thus, we think that the three classes of polyA sites do not influence each other.

**Figure 3:**
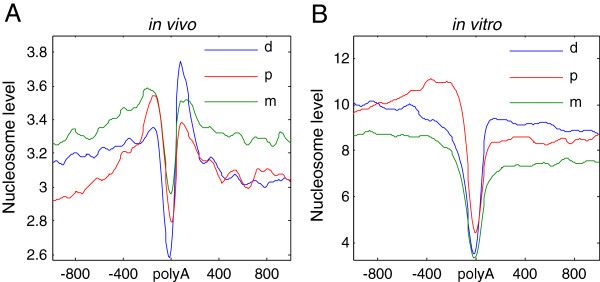
**Different patterns of nucleosome distribution around different alternative polyA sites. (A)** Nucleosome occupancy around distal (d), in-between (m) and proximal (p) polyA sites in human CD4+ T cells (*in vivo*). **(B)** Nucleosome occupancy around distal (d), in-between (m) and proximal (p) polyA sites *in vitro*.

We observed different nucleosome occupancy patterns between different types of alternative polyA sites, especially, the proximal and distal sites (Figure 3A). The proximal sites had higher upstream nucleosome occupancy than downstream nucleosome occupancy. The distal sites had better positioned downstream nucleosomes than upstream ones, which is similar to the constitutive polyA sites *in vivo*. The in-between sites were in between the two and their pattern was similar to that of the proximal sites. Compared with distal polyA sites, the nucleosome levels near and upstream of proximal polyA sites were significantly higher (*p* < 1 × 10^-11^, Figure 3A), whereas nucleosomes downstream of distal polyA sites were significantly higher than those downstream of proximal ones (*p* < 1 × 10^-15^, Figure 3A). Interestingly, these patterns of nucleosome distribution were reproduced in different cell types, *in vitro* and in prediction experiments (Figure 3B, Additional file 6), indicating an important contribution of nucleotide composition to the nucleosome occupancy pattern, suggesting that the nucleosome occupancy upstream of proximal sites and downstream of distal sites is inherently high.

Through quantitative analysis of physical properties of the nucleosome peak called by the DANPOS algorithm in CD4+ T cells, we confirmed that nucleosomes downstream of distal sites have a more consistent summit location and lower fuzziness score than those downstream of proximal sites (Additional file 2). Therefore, we concluded that nucleosomes immediately downstream of distal polyA sites are intrinsically better positioned. On the other hand, proximal polyA sites had more consistent positioning of upstream nucleosomes and higher nucleosome occupancy, suggesting that there are different patterns of nucleosome occupancy around proximal and distal sites, which may regulate polyadenylation and transcription termination by different mechanisms.

### Different regulation roles of nucleosome occupancy in the usage of alternative polyadenylation sites

**Figure 4:**
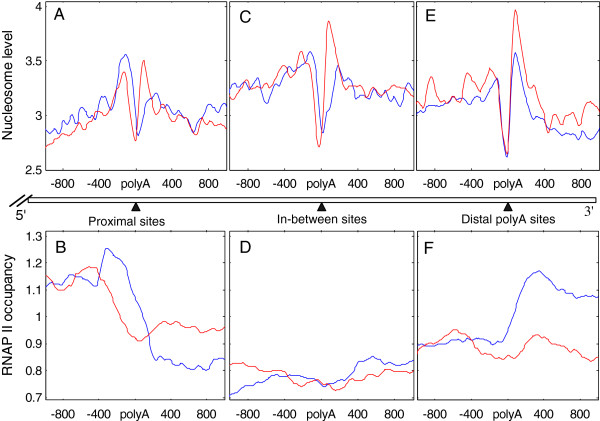
**Relationship among nucleosome, RNAP II occupancy and the usage of proximal, in-between and distal polyA sites. (A-B)** Nucleosome distribution and RNAP II occupancy near high-usage and low-usage proximal polyA sites of expressed genes in CD4+ T cell (RPKM > 1). **(C-D)** Nucleosome distribution and RNAP II occupancy near high-usage and low-usage in-between polyA sites of expressed genes in CD4+ T cell (RPKM > 1). **(E-F)** Nucleosome distribution and RNAP II occupancy near high-usage and low-usage distal polyA sites of expressed genes in CD4+ T cell (RPKM > 1). The blue curve represents high-usage sites that have the lowest RUD in the gene, and the red curve represents low-usage sites that have the highest RUD in the gene.

To explore the different regulatory effects of nucleosome occupancy on alternative polyadenylation and transcription termination, the relationship between the usage of proximal, distal and in-between polyA sites and nucleosome occupancy was analyzed using RNA-seq datasets for different cells. In expressed genes (RPKM > 1), the alternative polyA sites were divided into high-usage ones, with the lowest RUD in the gene, and low-usage ones, with the highest RUD in the gene. In the case of high-usage and low-usage distal polyA sites, the results showed that the varying tendencies of upstream and downstream nucleosome occupancy were the same, though the change in the upstream nucleosome level was smaller than that in the downstream nucleosome level (upstream nucleosome *p* < 1 × 10^-3^, downstream nucleosome *p* < 1 × 10^-19^; Figure 4E). The results also indicated that the varying tendencies of nucleosome occupancy were similar in different cells (Additional file 7A-B). RNAP II accumulated in the immediate downstream region of high-usage distal sites, while the downstream nucleosome level was significantly lower compared with the low-usage distal sites (Figure 4E-F). This suggests that nucleosome occupancy is associated with transcriptional activation. These observations are also consistent with constitutive polyA sites. Therefore, we concluded that nucleosomes downstream of distal polyA sites are similar to those downstream of constitutive sites and have a similar influence on the regulation the usage of polyA sites and transcription termination.

In proximal polyA sites, the change in downstream nucleosome occupancy was in good agreement with the change around the distal and constitutive sites, namely, the high-usage sites have significantly lower nucleosome occupancy than the low-usage sites (Figure 4A, Additional file 7C-D). Furthermore, one of the most significant findings in our study is that compared with the low-usage proximal sites, the high-usage proximal sites had a higher upstream nucleosome occupancy (*p* < 1 × 10^-11^, Figure 4A). It was further determined by quantitative analysis of nucleosome peak physical properties that high-usage proximal polyA sites had better positioned upstream nucleosomes (Figure 4A; high-usage Summit position = 97.4 ± 61.48, low-usage Summit position = 105.1 ± 65.75, *F* test *p* < 0.01 and Student’s *t* test *p* < 10^-4^). The nucleosome profile of high-usage proximal polyA sites shifted downstream compared with that of the low-usage sites. Meanwhile, RNAP II accumulated in the immediate upstream region of proximal polyA sites, and high-usage proximal polyA sites had a higher RNAP II occupancy than the low-usage sites had (Figure 4B). Furthermore, the RNAP II shifts were largely accompanied by coherent shifts in nucleosome occupancy. In in-between polyA sites, the nucleosome level was regulated by transcription and correlated with the usage of polyA sites (Figure 4C). However, the RNAP II occupancy was low near in-between polyA sites regardless of polyA site usage (Figure 4D).

**Figure 5:**
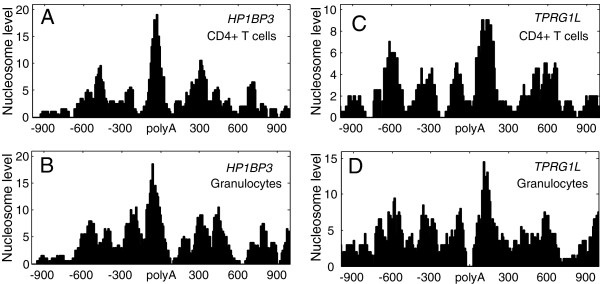
**Nucleosome occupancy surrounding proximal polyA sites of *HP1BP3 *and *TPRG1L *genes.***HP1BP3* and *TPRG1L* are highly expressed genes in both granulocytes and CD4+ T cells (RPKM > 20). Nucleosome occupancy around the proximal polyA site of *HP1BP3* which is highly used in both CD4+ T cells **(A)** and granulocytes **(B)** (Hs.142442.1.44 chr1 – 20975718), and around the proximal polyA site of *TPRG1L* which is lowly used in both CD4+ T cells **(C)** and granulocytes **(D)** (Hs.20529.1.8 chr1 + 3535226).

These observations imply that proximal polyA sites may have better positioned upstream nucleosomes to slow down RNAP II speed and improve polyA sites’ utilization. Figure 5 shows nucleosome-mapping data of two genes with high-usage proximal polyA sites or low-usage sites in both granulocytes and CD4+ T cells. This mapping indicates that nucleosomes downstream of proximal polyA sites are intrinsically better positioned and are expelled by RNAP II occupancy when the gene is transcribed, and that nucleosomes upstream of proximal sites are adjustable to change in the usage of the proximal sites and mark the beginning of transcription termination.

## Discussion

**Figure 6:**
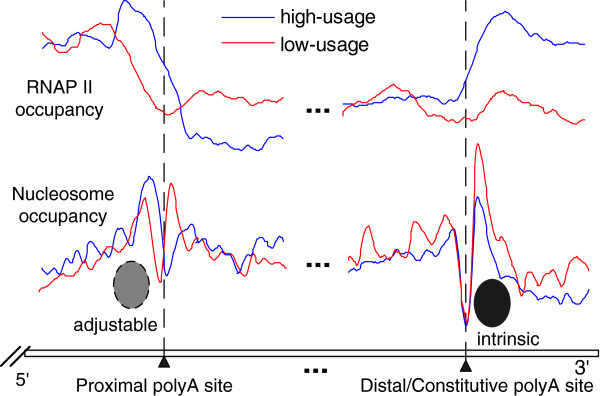
**The dual pausing model of RNAP II and nucleosome occupancy patterns during regulation of the usage of different polyA sites and transcription termination.** A nucleosome upstream of a proximal site, which is adjustable and related to the site usage, is the first barrier pausing RNAP II. Meanwhile, it signals to RNAP II to prepare for a 3′ end processing event, by slowing down transcription speed. A nucleosome downstream of distal or constitutive polyA site, which is intrinsically well positioned and regulated by transcription, is the second barrier pausing RNAP II to ensure transcription termination and recognition of the last polyadenylation sites if previous sites were missed.

Alternative polyadenylation is one of the mechanisms in human cells that give rise to a variety of transcripts from a single gene. More than half of the human genes have multiple polyadenylation sites, leading to various mRNA and protein products [26]. Epigenetic features and chromatin structure have been shown to be an important determinant of polyA site usage [14,15,20]. Here, we investigated the patterns of nucleosome distribution around different types of polyA sites of protein-coding genes in the human genome, and combined mRNA-seq and RNAP II datasets to uncover the regulation mechanism of nucleosome positioning in alternative polyadenylation and transcription termination. Genome-wide mapping of nucleosome occupancy *in vivo* and *in vitro* revealed that different nucleosome occupancy patterns correlate with the usage of different polyA sites and transcription termination (Summarized in Figure 6).

The current study demonstrated that compared with alternative polyA sites, a constitutive polyA site, defined as one polyA site in a gene, had better positioned downstream nucleosomes *in vivo* and *in vitro*, which was also demonstrated by predicted nucleosome positioning based on DNA sequence. Highly expressed genes had lower nucleosome occupancy near polyA sites than lowly expressed genes had. Meanwhile, RNAP II enrichment downstream of polyA sites was more pronounced than upstream. Thus, our results suggest that constitutive polyA sites have a greater intrinsic downstream nucleosome occupancy, which affects transcription termination by slowing down RNAP II to guarantee recognition of the only polyA site and effective transcription termination.

When examining alternative polyA sites, we found that nucleosome occupancy correlates with the sites’ position. Distal polyA sites, positioned closer to the end of the genes, had better positioned downstream nucleosomes than other alternative sites both *in vivo* and *in vitro*. Compared with low-usage distal polyA sites, highly used sites had lower nucleosome occupancy and higher RNAP II enrichment downstream of the polyA site, which is consistent with constitutive polyA sites. On the other hand, proximal polyA sites, positioned closer to the TSS of the genes, had better positioned upstream nucleosomes. Meanwhile, the pattern of nucleosome occupancy around in-between polyA sites was similar to the occupancy around proximal sites. High-usage proximal sites had higher upstream nucleosome occupancy and lower downstream nucleosome occupancy. Furthermore, there was a much greater accumulation of RNAP II in the high-usage class immediately upstream of the polyA site, which is very different from the patterns around distal and constitutive sites. We speculated that nucleosomes upstream of proximal polyA sites are adjustable, which correlates with polyA site usage.

Constitutive polyA sites can be taken as high-usage sites. Distal sites, favored in differentiated cells, exhibit a much higher frequency of canonical PAS and canonical DSE motif (likely stronger sites), and proximal polyA sites, favored in proliferating cells, tend to have limited consensus PAS features (likely weaker sites) [27]. We also found that distal polyA sites are more favored in granulocyte, CD8+ T cells and CD4+ T cells (Additional file 8). Thus, our results are in good agreement with a previous study showing that downstream nucleosomes correlate with the usage of polyA sites [20]. It has been shown that highly nucleosome occupancy downstream of polyA sites strongly correlated with a more favorable mRNA structure and greater accumulation of RNAP II at the polyA site [21], and improved the usage frequency of polyA sites. Furthermore, Grosso *et al*. have shown that nucleosome occupancy at the 3′ end of genes is dynamic and correlates with RNAP II density [28]. In addition, the results of Additional file 8 and Figure 4B can explain the phenomenon shown in Figure 2D. RNAP II accumulated immediately upstream of proximal polyA sites (Figure 4B). In low-usage alternative sites, proximal sites outnumber distal sites; almost triple the percentage of distal sites (Additional file 8). Thus, RNAP II accumulated immediately upstream of low-usage alternative polyA sites. Moreover, we observed that there was a little peak in the RNAP II profile upstream of high-usage alternative polyA sites (Figure 2D), which was attributed to the contribution of proximal sites of the high-usage alternative polyA sites.

Our results specifically point out that downstream nucleosomes correlate with the usage of constitutive and distal sites. Additionally, we inferred that nucleosomes downstream of constitutive and distal polyA sites are intrinsically better positioned, to guarantee stronger polyA site recognition and facilitate the formation of a stable RNA structure at the polyA site by reducing RNAP II transcription speed. Moreover, the nucleosome occupancy curve around constitutive and distal polyA sites was much steeper than other sites *in vivo*, which makes it more difficult for RNAP II to pass. Also, accumulation of RNAP II at the 3′ end of genes correlated with the usage of polyA sites. Thus, a possible role of downstream nucleosomes is to guarantee gene transcription termination and recognition of the last polyadenylation sites if previous sites were missed.

Proximal polyA sites have an intrinsic advantage over the distal ones, as they are transcribed earlier and have more time to be recognized (“first come, first served”), while higher elongation rate leads to them being missed and to enhance the recognition of the distal polyA sites [29]. According to the mechanisms of alternative polyadenylation regulation, the proximal polyA site may mainly serve as an alternative site. Hence, we inferred that nucleosomes upstream of polyA sites play regulatory roles that promote the recognition of weaker proximal sites by slowing down transcription speed and recruiting 3′-processing factors before RNAP II arrives at the polyA site. On the other hand, they may serve as the boundary of the polyA sites region, signaling to RNAP II to slow down and prepare for a 3′ end processing event. Such signaling may be necessary to reduce transcription speed in polyA + genes, where there is alternative polyadenylation. Furthermore, there was a nucleosome shift toward the 3′ end in highly used sites, which was largely accompanied by a coherent shift of RNAP II occupancy. We conjecture that this may be related to the dynamic mechanism of transcription through chromatin by RNAP II. Chromatin remodeling factors and histone chaperones dissociate from the transcription complex near the polyA site to help the polymerase remodel the nucleosome [30]. The increase in RNAP II residence time near a proximal/distal site may in turn reduce the amount of RNAP II that can be captured near in-between sites.

## Conclusions

Taken together, our study indicates that nucleosomes near polyA sites have various regulatory effects on polyadenylation and transcription termination (Figure 6). Proximal polyadenylation sites have an adjustable upstream nucleosome occupancy, which correlates with the site usage, and may signal to RNA polymerase II to slow down and prepare for a 3′ end processing event. Distal and constitutive sites have intrinsically well-positioned downstream nucleosomes, which are regulated by transcription and are the second barrier pausing RNAP II to ensure transcription termination and recognition of the last polyadenylation sites, if previous sites were missed. Nucleosome arrays around polyA sites play different roles in the regulation of the usage of polyadenylation sites and transcription termination of protein-coding genes, and form a dual pausing model of RNA polymerase II in the alternative polyadenylation sites’ region, to ensure effective 3′ end processing. Histone modification as the main epigenetic marker has been shown to regulate alternative splicing by affecting splicing regulators and recruiting the spliceosome [31]. It will be interesting to examine how different types of histone modifications affect different types of polyA site usage.

## Methods

### PolyA site dataset

The genomic coordinates of the polyA sites of the human genome were obtained from the PolyA_DB2 database [26]. We used the Batch Coordinate Conversion (liftOver) tool from the UCSC Genome Bioinformatics resource to remap the polyA sites from NCBI35/hg17 to NCBI36/hg18 (http://genome.ucsc.edu/cgi-bin/hgLiftOver) [32]. All polyA sites that were not uniquely mapped to the reference sequence of the protein-coding gene were removed. PolyA sites in genes with only one polyA site are called constitutive sites. PolyA sites in genes with multiple sites were alternative sites. According to the number of polyA sites in the gene, genes were divided into two classes: 8412 genes with a single constitutive polyA site and 9311 genes with alternative polyA sites.

In genes with alternative polyA sites, the sites were divided into proximal, distal and in-between polyA sites, according to the position of the polyA site relative to the TSS of the gene. The polyA sites closest to the TSS of a gene were proximal sites, the sites closest to the end of a gene were distal sites, and the polyA sites located between proximal and distal polyA sites were in-between sites. The coordinates of the TSS and the end of genes were obtained from the annotation of RefSeq genes in UCSC Table Browser (hg18 database, http://genome.ucsc.edu/cgi-bin/hgTables).

### MNase sequencing and RNAP II data and data analysis

Sequencing datasets of nucleosome positions in different human cell types (CD4+ T cells, CD8+ T cells and granulocytes) and *in vitro*, generated by high-throughput SOLiD technology, were analyzed [33]. Tag coordinate bed files of RNAP II in human CD4+ T cells using the Solexa sequencing technology were also used [34].

Nucleosome scores calculated as previously described [33], represent the average number of sequenced reads uniquely mapping to the sense strand 80 bases upstream and to the antisense strand 80 bases downstream of one locus with a step size of 10 bp. Nucleosome occupancy was calculated in alignment with polyA sites, and was smoothed using a moving average filter. The moving average span was 5. The Wilcoxon rank sum test was used to gauge the significance (*p*). RNAP II occupancy was calculated similarly to the nucleosome occupancy, but the score represents the average number of sequenced reads uniquely mapped to the sense strand 300 bases upstream and to the antisense strand 300 bases downstream of one locus.

Nucleosome occupancy peak calling was performed by the DANPOS algorithm [35], which was designed for dynamic nucleosome analysis at single-nucleotide resolution by sequencing and can transform reads data of each replicate to occupancy and provide peak summit position and peak fuzziness score.

### Analysis of gene expression levels and polyA site usage using mRNA-seq data

The mRNA-seq data performed on nuclear extracts from human CD4+ T cells, CD8+ T cells and granulocytes (accession number GSE25133) [33] were obtained from NCBI’s Gene Expression Omnibus. The sequencing reads were aligned using TopHat against human genome (hg18) [36], allowing at most two mismatches. Gene expression levels were quantified using read density in the protein-coding region based on the reads per kilobase of mappable region per million mapped reads (RPKM) method [37].

To evaluate polyA site usage, the density of reads mapped to upstream and downstream regions was compared, as illustrated in a previous study [38]. The relative usage of downstream polyA site (RUD) score was calculated, which is the ratio between the density of downstream reads and the density of upstream reads. A low RUD score for a polyA site represents high usage of the polyA site. The reads mapped in the ±10-nt region around the polyA sites were not used for RUD calculation, because usually the cleavage sites are not precise.

## Competing interests

The authors declare that they have no competing interests.

## Authors’ contributions

HH conceived the study and wrote the paper. HH, HDL and XS designed the bioinformatics analyses. JC collected and preprocessed data sets. All authors read and approved the final manuscript.

## Supplementary Material

Additional file 1 **Nucleosome distribution around constitutive and alternative polyA sites. (A-B)** Nucleosome occupancy surrounding polyA sites of constitutive (con) and alternative (alt) polyA sites across a 2000-bp window in granulocytes and CD8+ T cells. **(C)** Predicted nucleosome occupancy based on DNA sequence around constitutive (con) and alternative (alt) polyA sites across a 2000-bp window. Click here for file

Additional file 2 **Statistics of the summit position distribution and fuzziness score of nucleosome peaks called by the DANPOS algorithm within 300 bp upstream and 300 bp downstream of the polyA sites.** The standard deviation (std) of the distance between the summit position and polyA sites, and the average value of fuzziness score of nucleosome peaks were calculated to appraise the consistency of nucleosome positioning. The sample used for the analyses was CD4+ T cells shown in Figures 1A and 3A. Click here for file

Additional file 3 **The relationship between the nucleosome and the usage of polyA sites. (A-B)** Nucleosome distribution near constitutive polyA sites of highly expressed and lowly expressed genes in granulocytes and CD8+ T cells (highly expressed genes: RPKM > 10, blue curve; lowly expressed genes: RPKM < 0.1, red curve). **(C-D)** Nucleosome distribution near high-usage (blue curve) and low-usage (red curve) alternative polyA sites in expressed genes in granulocytes and CD8+ T cells (RPKM > 1). High-usage sites have the lowest RUD in the gene, and low-usage sites have the highest RUD in the gene. Click here for file

Additional file 4 **Nucleosome level around constitutive polyA sites of differentially expressed genes between CD4+ T cells and granulocytes.** The blue curve represents the genes that were highly expressed in CD4+ T cells (RPKM > 10) and unexpressed in granulocytes (RPKM < 0.1). The red curve represents the genes that were unexpressed in CD4+ T cells (RPKM < 0.1) and highly expressed in granulocytes (RPKM >10). Click here for file

Additional file 5 The average distance from the three classes of polyA sites to the TSS and to the end of the gene. Click here for file

Additional file 6 **Different patterns of nucleosome distribution around different alternative polyA sites. (A-B)** Nucleosome occupancy around distal (d), in-between (m) and proximal (p) polyA sites across a 2000-bp window in granulocytes and CD8+ T cells. **(C)** Predicted nucleosome occupancy based on DNA sequence around distal (d), in-between (m) and proximal (p) polyA sites. Click here for file

Additional file 7 **Relationship between nucleosomes and the usage of proximal and distal polyA sites. (A-B)** Nucleosome occupancy surrounding high-usage and low-usage distal polyA sites of expressed genes (RPKM > 1) in granulocytes and CD8+ T cells. **(C-D)** Nucleosome occupancy surrounding high-usage and low-usage proximal polyA sites of expressed genes (RPKM > 1) in granulocytes and CD8+ T cells. The blue curve represents high-usage sites that have the lowest RUD in the gene; and the red curve represents low-usage sites that have the highest RUD in the gene. Click here for file

Additional file 8 **Percentage of distal and proximal polyA sites in high-usage and low-usage alternative polyA sites.** The percentage of distal (distal) and proximal (proximal) polyA sites in high-usage and low-usage alternative polyA sites in granulocytes **(A)**, CD4+ T cells **(B)** and CD8+ T cells **(C)**. High-usage polyA sites have the lowest RUD in expressed genes (RPKM > 1), and low-usage polyA sites have the highest RUD in expressed genes (RPKM > 1). Click here for file
