# Supplementary figures and images for: The nucleosome regulates the usage of polyadenylation sites in the human genome
Source: BMC Genomics. 2013 Dec 23;14:912. doi: 10.1186/1471-2164-14-912 (PMC3879661; doi:10.1186/1471-2164-14-912)

## Slide 1
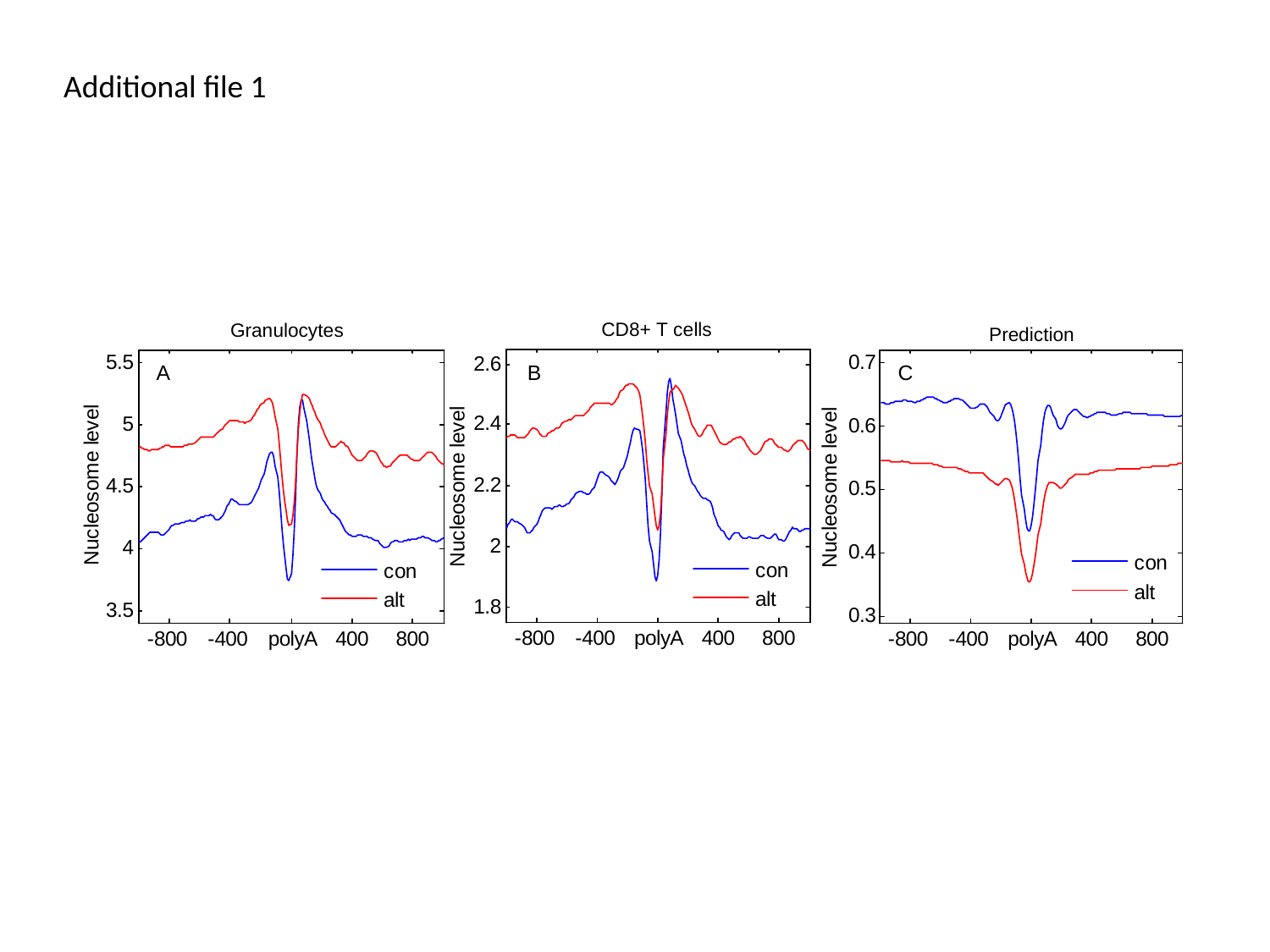

Additional file 1

Supplement: Additional file 1 — Nucleosome distribution around constitutive and alternative polyA sites. (A-B) Nucleosome occupancy surrounding polyA sites of constitutive (con) and alternative (alt) polyA sites across a 2000-bp window in granulocytes and CD8+ T cells. (C) Predicted nucleosome occupancy based on DNA sequence around constitutive (con) and alternative (alt) polyA sites across a 2000-bp window. [file 1471-2164-14-912-S1.ppt]

## Slide 1
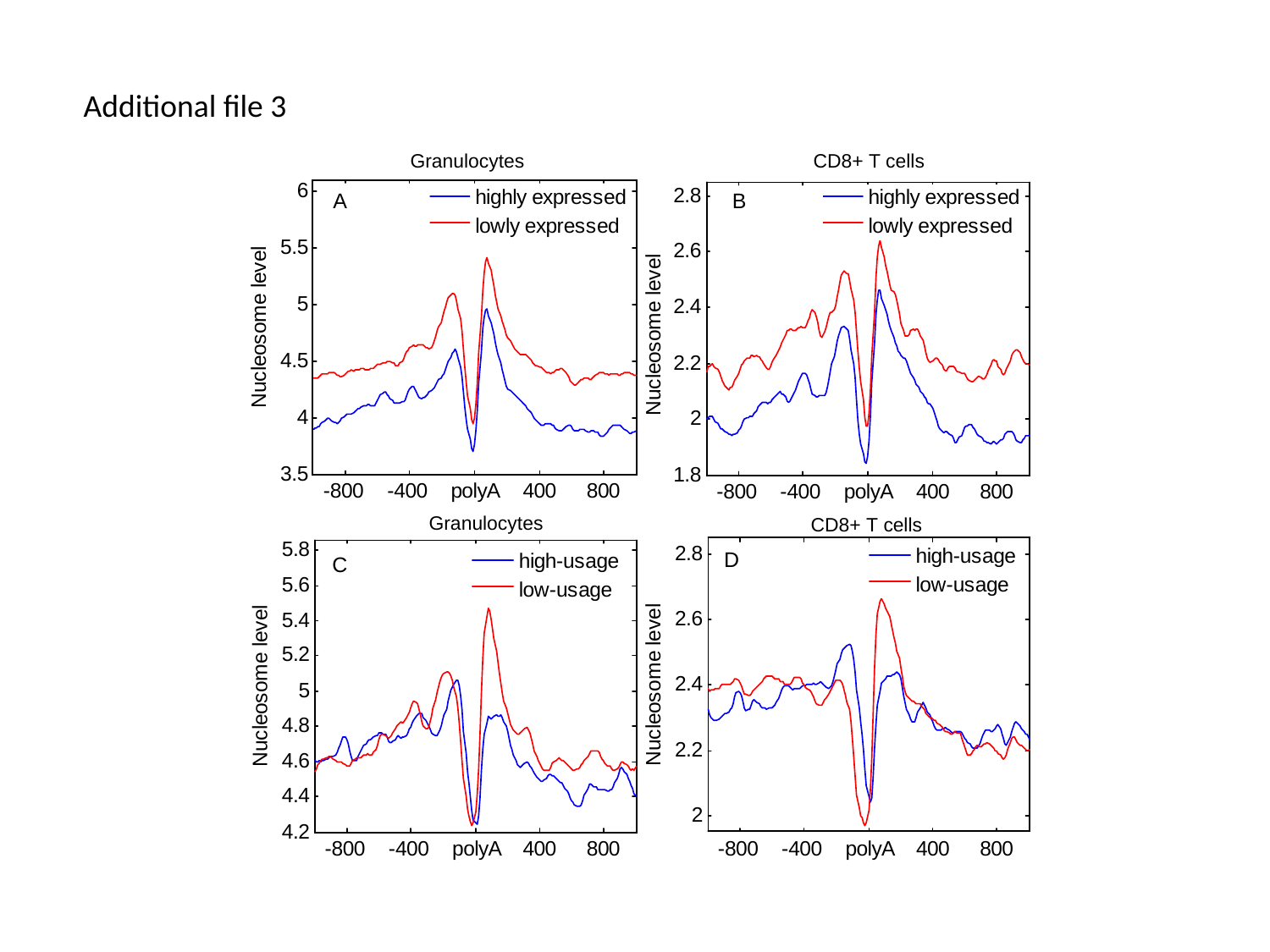

Additional file 3

Supplement: Additional file 3 — The relationship between the nucleosome and the usage of polyA sites. (A-B) Nucleosome distribution near constitutive polyA sites of highly expressed and lowly expressed genes in granulocytes and CD8+ T cells (highly expressed genes: RPKM > 10, blue curve; lowly expressed genes: RPKM < 0.1, red curve). (C-D) Nucleosome distribution near high-usage (blue curve) and low-usage (red curve) alternative polyA sites in expressed genes in granulocytes and CD8+ T cells (RPKM > 1). High-usage sites have the lowest RUD in the gene, and low-usage sites have the highest RUD in the gene. [file 1471-2164-14-912-S3.ppt]

## Slide 1
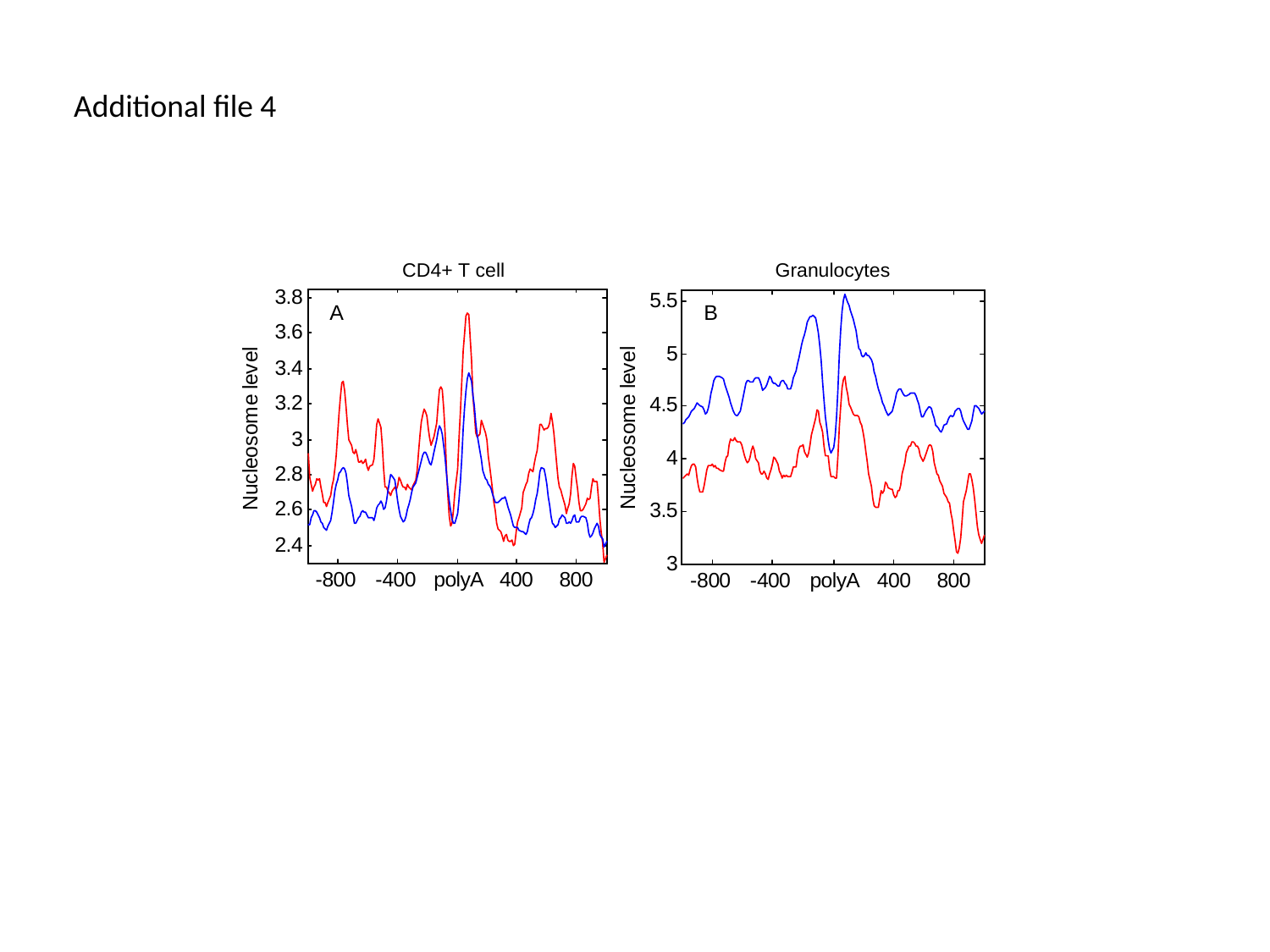

Additional file 4

Supplement: Additional file 4 — Nucleosome level around constitutive polyA sites of differentially expressed genes between CD4+ T cells and granulocytes. The blue curve represents the genes that were highly expressed in CD4+ T cells (RPKM > 10) and unexpressed in granulocytes (RPKM < 0.1). The red curve represents the genes that were unexpressed in CD4+ T cells (RPKM < 0.1) and highly expressed in granulocytes (RPKM >10). [file 1471-2164-14-912-S4.ppt]

## Slide 1
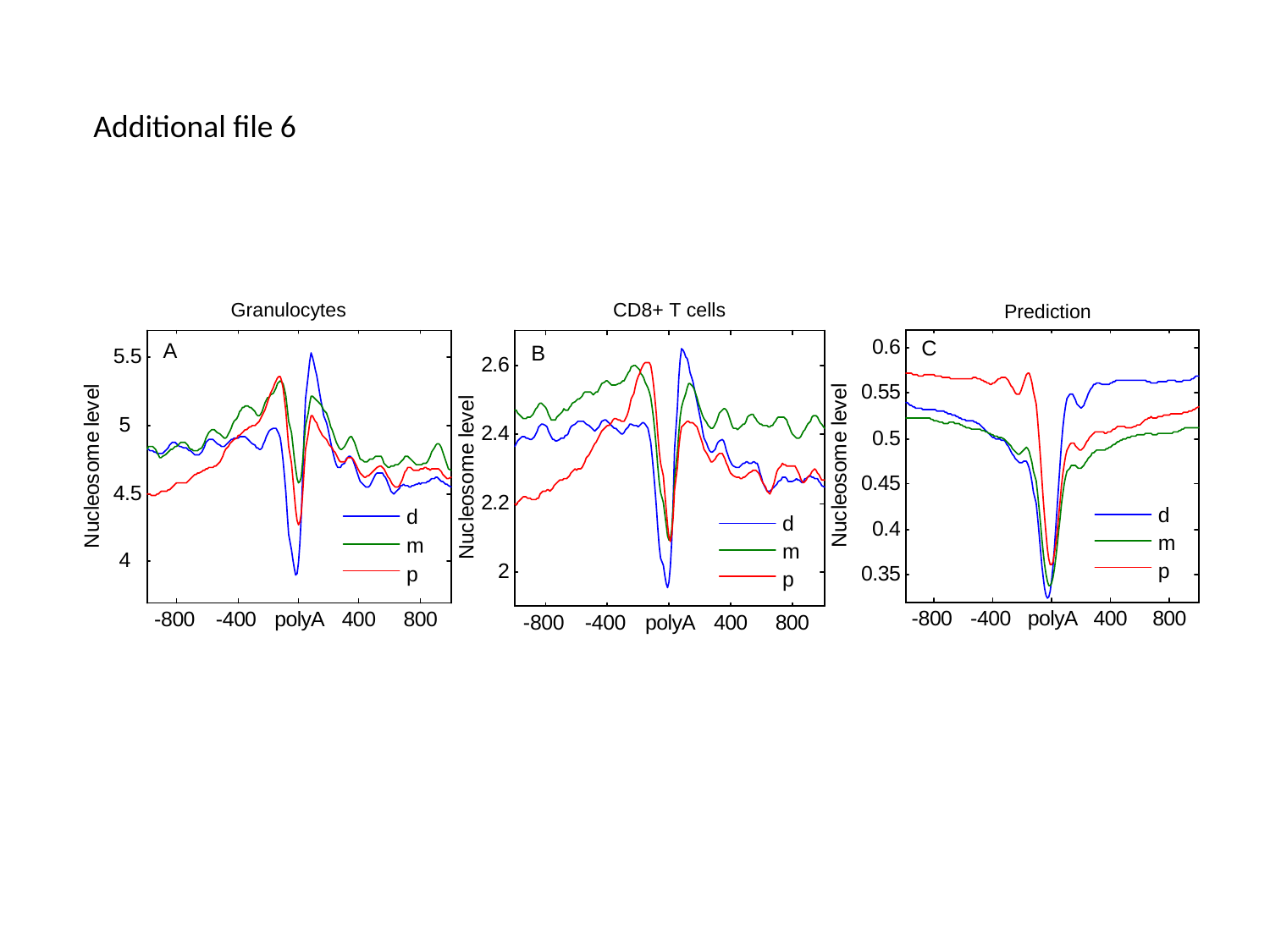

Additional file 6

Supplement: Additional file 6 — Different patterns of nucleosome distribution around different alternative polyA sites. (A-B) Nucleosome occupancy around distal (d), in-between (m) and proximal (p) polyA sites across a 2000-bp window in granulocytes and CD8+ T cells. (C) Predicted nucleosome occupancy based on DNA sequence around distal (d), in-between (m) and proximal (p) polyA sites. [file 1471-2164-14-912-S6.ppt]

## Slide 1
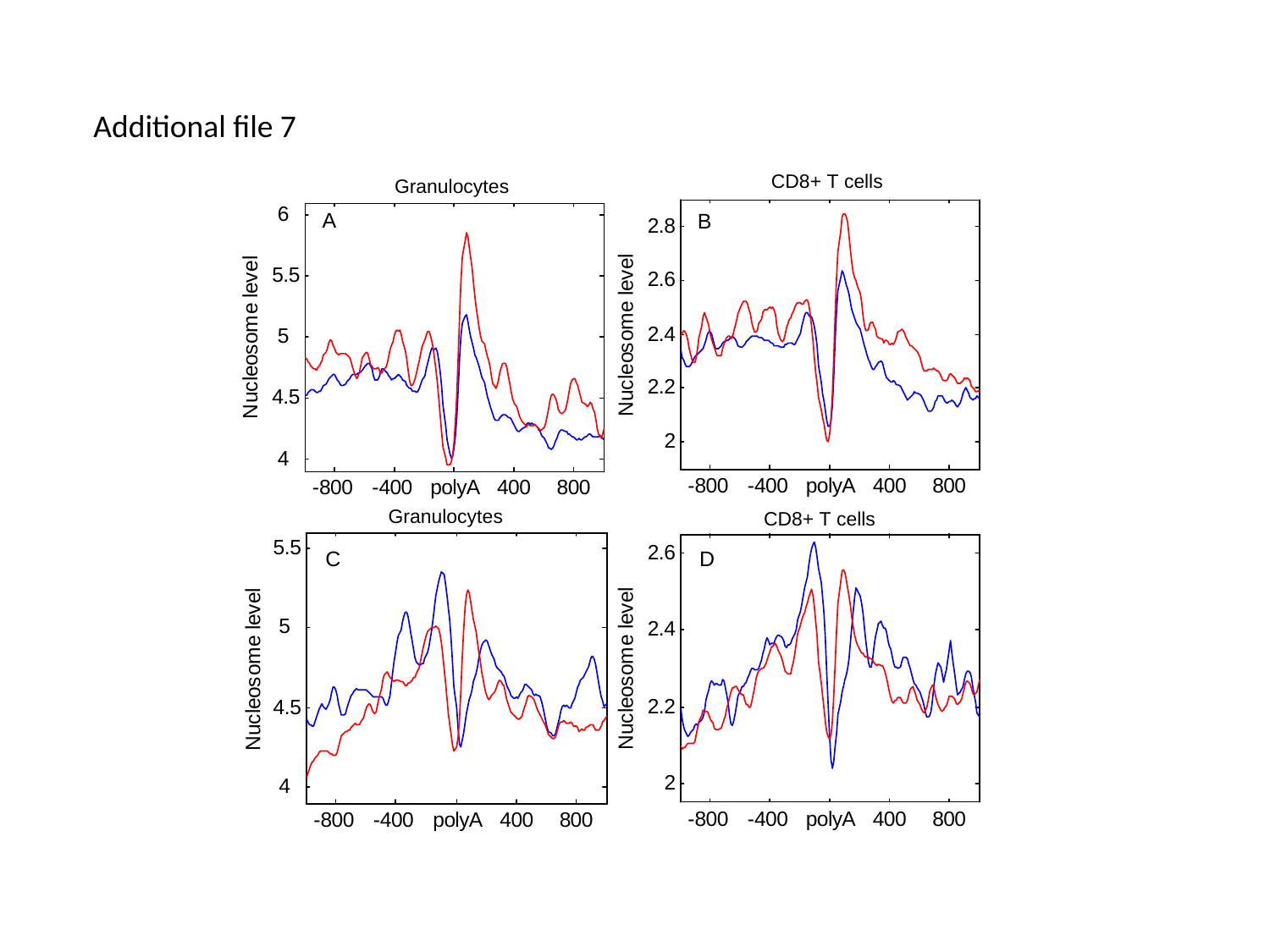

Additional file 7

Supplement: Additional file 7 — Relationship between nucleosomes and the usage of proximal and distal polyA sites. (A-B) Nucleosome occupancy surrounding high-usage and low-usage distal polyA sites of expressed genes (RPKM > 1) in granulocytes and CD8+ T cells. (C-D) Nucleosome occupancy surrounding high-usage and low-usage proximal polyA sites of expressed genes (RPKM > 1) in granulocytes and CD8+ T cells. The blue curve represents high-usage sites that have the lowest RUD in the gene; and the red curve represents low-usage sites that have the highest RUD in the gene. [file 1471-2164-14-912-S7.ppt]

## Slide 1
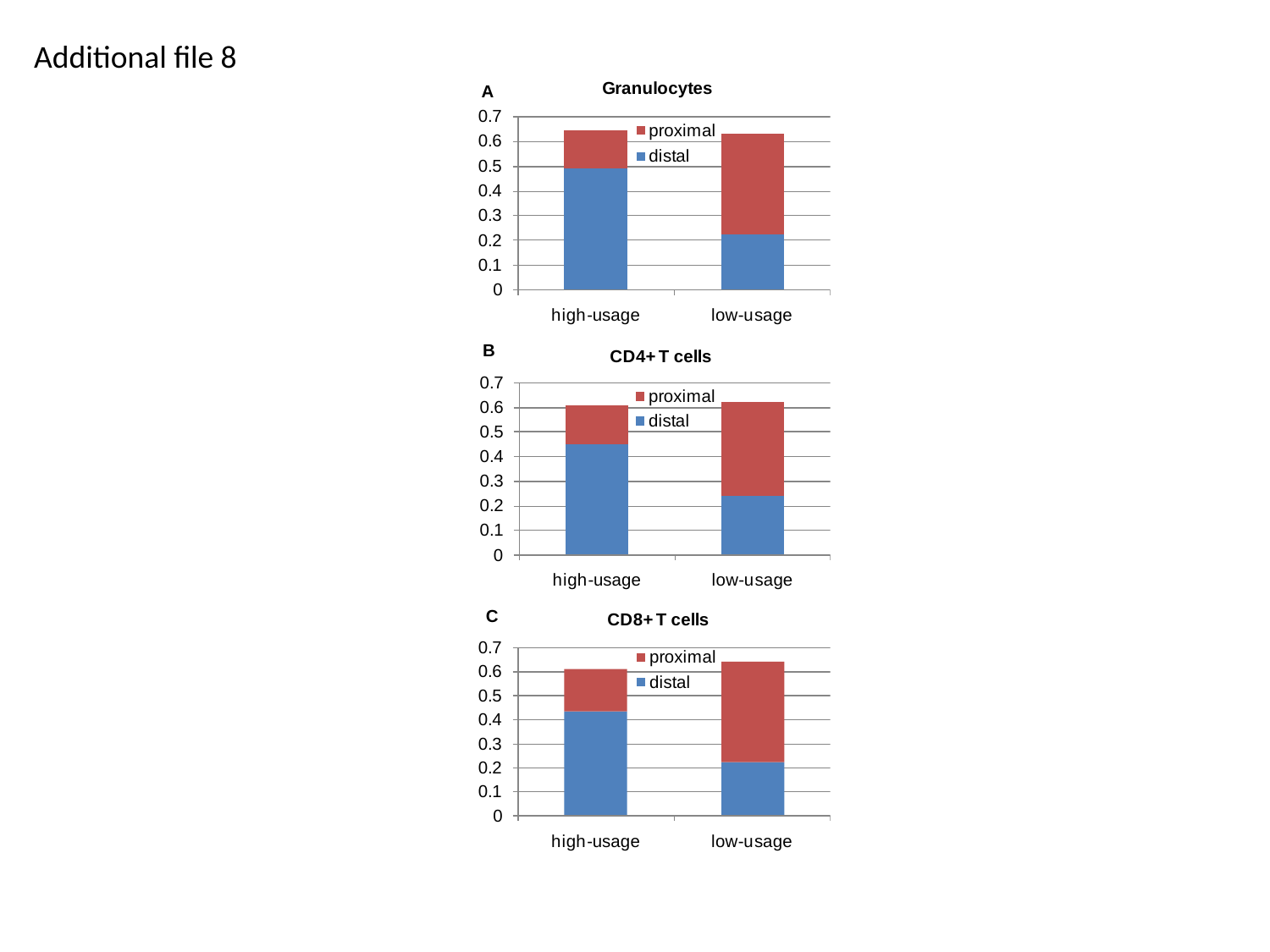

Additional file 8

Supplement: Additional file 8 — Percentage of distal and proximal polyA sites in high-usage and low-usage alternative polyA sites. The percentage of distal (distal) and proximal (proximal) polyA sites in high-usage and low-usage alternative polyA sites in granulocytes (A), CD4+ T cells (B) and CD8+ T cells (C). High-usage polyA sites have the lowest RUD in expressed genes (RPKM > 1), and low-usage polyA sites have the highest RUD in expressed genes (RPKM > 1). [file 1471-2164-14-912-S8.ppt]
