# Supplementary material for: The nucleosome regulates the usage of polyadenylation sites in the human genome
Source: BMC Genomics. 2013 Dec 23;14:912. doi: 10.1186/1471-2164-14-912 (PMC3879661; doi:10.1186/1471-2164-14-912)
Supplement: Additional file 2 — Statistics of the summit position distribution and fuzziness score of nucleosome peaks called by the DANPOS algorithm within 300 bp upstream and 300 bp downstream of the polyA sites. The standard deviation (std) of the distance between the summit position and polyA sites, and the average value of fuzziness score of nucleosome peaks were calculated to appraise the consistency of nucleosome positioning. The sample used for the analyses was CD4+ T cells shown in Figures 1A and 3A. [file 1471-2164-14-912-S2.ppt]

## Slide 1
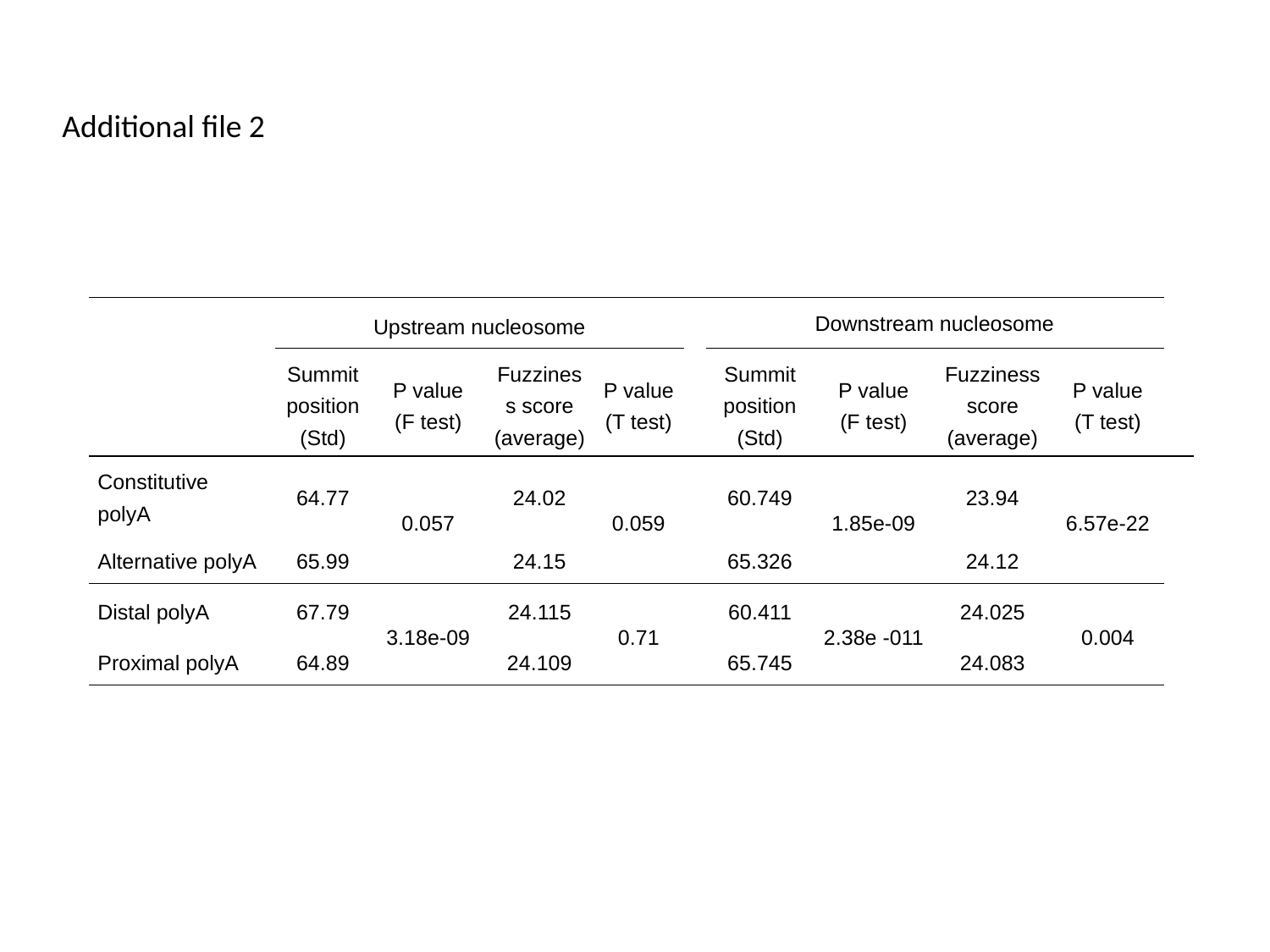

Additional file 2
| | Upstream nucleosome | | | | | Downstream nucleosome | | | | |
| --- | --- | --- | --- | --- | --- | --- | --- | --- | --- | --- |
| | Summit position (Std) | P value (F test) | Fuzziness score (average) | P value (T test) | | Summit position (Std) | P value (F test) | Fuzziness score (average) | P value (T test) | |
| Constitutive polyA | 64.77 | 0.057 | 24.02 | 0.059 | | 60.749 | 1.85e-09 | 23.94 | 6.57e-22 | |
| Alternative polyA | 65.99 | | 24.15 | | | 65.326 | | 24.12 | | |
| Distal polyA | 67.79 | 3.18e-09 | 24.115 | 0.71 | | 60.411 | 2.38e -011 | 24.025 | 0.004 | |
| Proximal polyA | 64.89 | | 24.109 | | | 65.745 | | 24.083 | | |
