# Supplementary material for: The nucleosome regulates the usage of polyadenylation sites in the human genome
Source: BMC Genomics. 2013 Dec 23;14:912. doi: 10.1186/1471-2164-14-912 (PMC3879661; doi:10.1186/1471-2164-14-912)
Supplement: Additional file 5 — The average distance from the three classes of polyA sites to the TSS and to the end of the gene. [file 1471-2164-14-912-S5.ppt]

## Slide 1
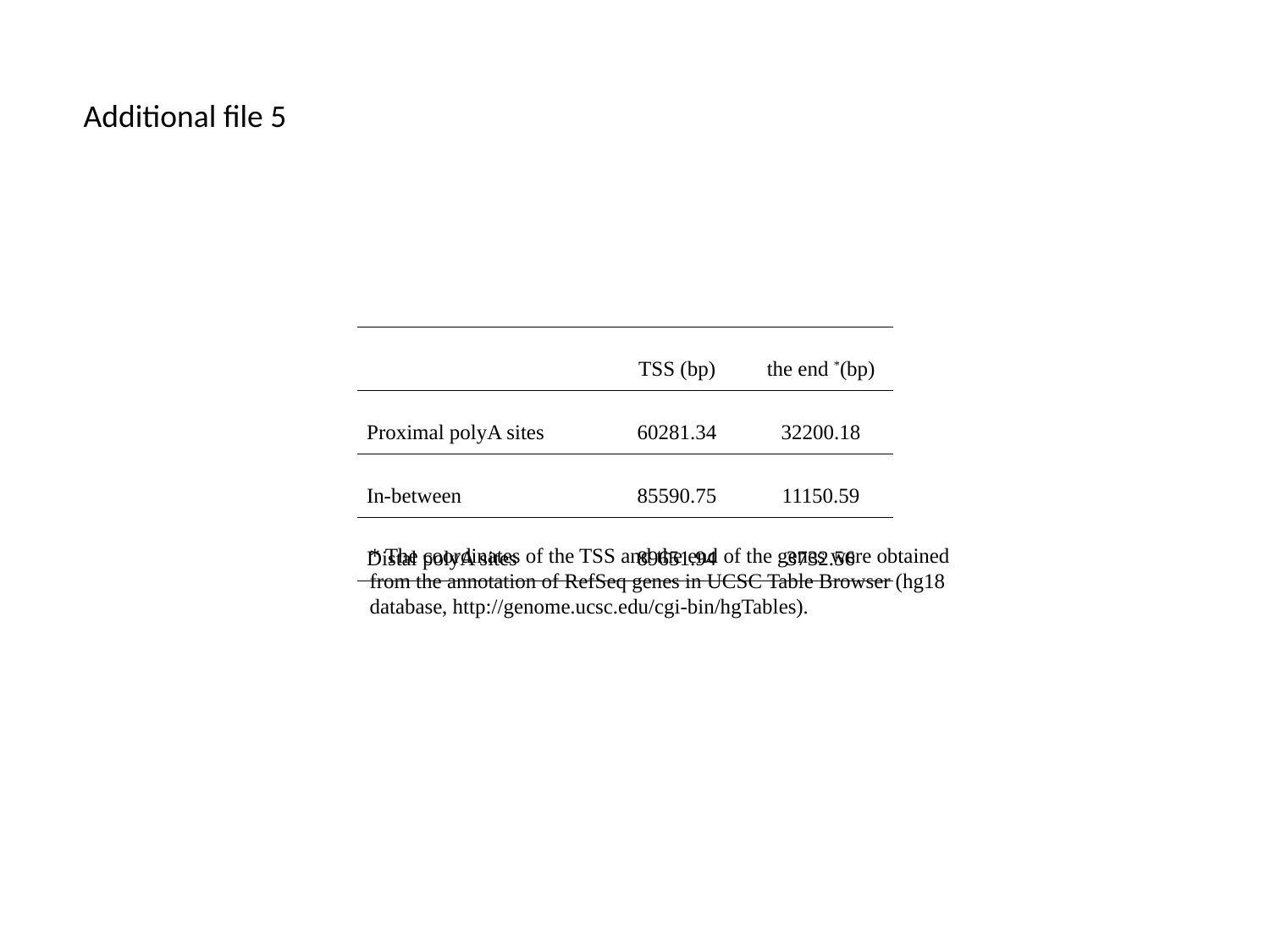

Additional file 5
| | TSS (bp) | the end \*(bp) |
| --- | --- | --- |
| Proximal polyA sites | 60281.34 | 32200.18 |
| In-between | 85590.75 | 11150.59 |
| Distal polyA sites | 89651.94 | 3732.56 |
* The coordinates of the TSS and the end of the genes were obtained from the annotation of RefSeq genes in UCSC Table Browser (hg18 database, http://genome.ucsc.edu/cgi-bin/hgTables).
